# Environmental and microbial factors influence affective and cognitive behavior in C57BL/6 sub-strains

**DOI:** 10.3389/fimmu.2023.1139913

**Published:** 2023-04-25

**Authors:** Nada Abdel Aziz, Inssaf Berkiks, Paballo Mosala, Tiroyaone M. Brombacher, Frank Brombacher

**Affiliations:** ^1^ Cytokine and Disease Group, International Centre for Genetic Engineering and Biotechnology, Cape Town Component, Division of Immunology, Institute of Infectious Diseases and Molecular Medicine, Faculty of Health Sciences, University of Cape Town, Cape Town, South Africa; ^2^ Immuno-Biotechnology Group, Biotechnology Department, Faculty of Science, Cairo University, Cairo, Egypt; ^3^ Wellcome Centre for Infectious Diseases Research in Africa, Institute of Infectious Diseases and Molecular Medicine (IDM), Faculty of Health Sciences, University of Cape Town, Cape Town, South Africa

**Keywords:** C57BL/6 sub-strains, microbiome, environmental factor, cognitive function, affective behavior, immune cells, meninges, hippocampus

## Abstract

C57BL/6 mice are one of the most widely used inbred strains in biomedical research. Early separation of the breeding colony has led to the development of several sub-strains. Colony separation led to genetic variation development driving numerous phenotypic discrepancies. The reported phenotypic behavior differences between the sub-strains were, however; not consistent in the literature, suggesting the involvement of factors other than host genes. Here, we characterized the cognitive and affective behavior of C57BL/6J and C57BL/6N mice in correlation with the immune cell profile in the brain. Furthermore, faecal microbiota transfer and mice co-housing techniques were used to dissect microbial and environmental factors’ contribution, respectively, to cognitive and affective behavior patterns. We first noted a unique profile of locomotor activity, immobility pattern, and spatial and non-spatial learning and memory abilities between the two sub-strains. The phenotypic behavior profile was associated with a distinct difference in the dynamics of type 2 cytokines in the meninges and brain parenchyma. Analysing the contribution of microbiome and environmental factors to the noted behavioral profile, our data indicated that while immobility pattern was genetically driven, locomotor activity and cognitive abilities were highly sensitive to alterations in the gut microbiome and environmental factors. Changes in the phenotypic behavior in response to these factors were associated with changes in immune cell profile. While microglia were highly sensitive to alteration in gut microbiome, immune cells in meninges were more resilient. Collectively, our findings demonstrated a direct impact of environmental conditions on gut microbiota which subsequently impacts the brain immune cell profile that could modulate cognitive and affective behavior. Our data further highlight the importance of characterizing the laboratory available strain/sub-strain to select the most appropriate one that fits best the study purpose.

## Introduction

C57BL/6 mouse model is one the most widely used strains in biomedical sciences. This strain has been created from C57BL line by Dr. C.C. Little in 1920 (1, 2). Through the separation of the breeding colonies, two main sub-strains have been developed which are C57BL/6J and C57BL/6N. While the former has been maintained in Jackson's laboratory, the latter is in the National Institute of Health (NIH). Several other sub-strains have then been derived from the two main lineages and kept by different vendors in independent facilities (1, 2). While all the sub-strains do share the same phenotypical appearance, they are not genetically identical (1, 3). Over the years, genetic shift and drift have led to the development of several single nucleotide polymorphisms, indels, and structural variants in these sub-strains (3, 4).

Genetic shift and drift have led to considerable variations in several phenotypic traits between the sub-strains. For instance, it has been shown that C57BL/6J and C57BL/6N differ in their susceptibility to ocular lesions development (5), hypothermia-induced seizure (6), drug abuse response (7), and in the maintenance of glucose homeostasis and insulin secretion (8). Differences in phenotypic behavior including fear, anxiety, and pain were also reported (2–4, 9–11). These alterations were, however; not consistent in the literature (2–4, 9–11). Recent studies have indicated that phenotypic behavior inconsistency could be explained by the fact that some phenotypes are not solely derived by host genetics, but rather through alteration in microbial profile and/or interaction between host and microbial factors (12–14). In fact, there is an interplay between host genetics, environmental factors, microbiome composition, and phenotype development (13). Thus, variation in any of these factors can lead to manifestation of different phenotypes. For instance, alteration in environmental factors can result in changes in microbial composition and thus the microbial factors available in the milieu that can subsequently impact host biology and functionality (13, 14). Whether the variation in environmental factors and thus microbiome composition could account for some of the documented behavioral differences between C57BL/6 sub-stains is an open question that is yet to be experimentally addressed. Furthermore, it is not yet clear how genetic, environmental, and microbial factors affect brain immune cell profile and thus, brain functionality in these sub-strains.

In the present study, we assessed the pattern of affective and cognitive behavior in C57BL/6J (B6J) and C57BL/6N (B6N) mice in association with immune cell profile in meninges and brain parenchyma. Using gut microbiota transfer and environmental co-housing, we investigated the extent gut microbiome and environmental factors contribute to cognitive and affective behavior patterns. We further dissected the impact of these factors on the responsiveness of immune cells in meninges and brain parenchyma.

## Material and methods

### Ethics statement and mice

All mice were maintained in specific-pathogen-free barrier conditions in individually ventilated cages at the University of Cape Town biosafety level 2 animal unit facility. C57BL/6J and C57BL6/N mice were maintained in two different SPFs facilities. C57BL/6N mice were maintained in SPF that is free of all FELASA-listed organisms except for *Pasteurella pneumotropica*, *Helicobacter* spp. and *murine norovirus* (MNV). The C57BL/6J mice were maintained in SPF facility that is free of all FELASA-listed organisms. Experimental mice were sex and age-matched and used at 12 weeks of age. Mice were maintained in IVC cages with the dimension of 330 mm x 170 mm x 140 mm (lxbxh) on a wood shaving bedding. All the experimental work was in strict accordance with the recommendations of the South African national guidelines and of the University of Cape Town practice for laboratory animal procedures as in ethics protocols, 020-007 and 020-002, approved by the Animal Research Ethics Committee of the Faculty of Health Sciences, University of Cape Town. All efforts were made to minimize animal suffering.

### Antibiotic treatment and faecal microbiota transfer

Mice were first habituated for a week and then treated for five consecutive days with an antibiotic cocktail followed by FMT transfer for 3 days as in (15). Briefly, mice were treated with ampicillin (2 mg/ml), neomycin (2 mg/ml), metronidazole (2 mg/ml), and vancomycin (1 mg/ml) *via* oral gavage for five consecutive days. Frozen faecal pellets collected from donor mice were reconstituted in sterile ice-cold PBS in a concentration of 100 mg/ml. The pellets were homogenised, spun down at 800 xg for 3 mins, and the supernatant was separated for oral gavage treatment for 3 consecutive days immediately after antibiotic treatment. Mice were then exposed to a battery of affective and cognitive function tests.

### Co-housing experiment

Mice were received at 12 weeks of age. After allowing one week to habituate in the biosafety level 2 facility, C57BL/6J and C57BL/6N mice were placed in clean cages (with new food and water) at a ratio of 1:1 and co-housed for six months. Mice were monitored for any signs of fighting (i.e wounds, tail lesions, or any injuries) to ensure that there is no fighting between the two strains. Ear punching facilitated long-term identification (16, 17). At the end of the co-housing period, mice were exposed to a battery of behavioral tests for assessing cognitive and affective behavior.

### Behavioral testing

In each experimental set, cognitive and affective behavior were evaluated using a comprehensive behavioral battery starting with the least to the most stressful ones. Mice were exposed to open field, then novel object, light-dark, and finally forced swim test.

### Open field test

The open field test is usually used to assess locomotor activity, avoidance behavior, and anxiety-like behavior in animals (18). In this test, each mouse was placed in an open field apparatus (30 cm x 30 cm x 32 cm) under strong illumination (60 lux lighting). The open field’s area was then virtually divided into central and peripheral area. After mice acclimatization, each mouse was placed in the centre of the apparatus to explore the arena for 10 mins. EthoVision^®^ XT 8 automated tracking system (Noldus Information Technology, VA) was used for video tracking and data analysis.

### Light-dark box test

The light/dark box test is a widely used tests to assess anxiety-like behavior in mice. The test is based on the natural aversion and preference of mice to react to brightly illuminated areas and on their spontaneous exploratory behavior in novel environments (19). The apparatus was divided into two chambers: a dark chamber and a bright chamber. Mice were placed in the light compartment (1:1) and behavior was recorded for 10 min. Measurements of total time spent in the light versus dark compartments and the number of transitions from one compartment to the other were recorded using EthoVision^®^ XT 8.

### Forced swim test

FST is a test used to assess depression-like behavior. In this test, swimming sessions were conducted by placing the mouse in individual glass cylinders (30 cm in height and 20 cm in diameter) containing 30 cm of water at (23 ± 2 °C). During the session, mice were forced to swim for 6 min and the duration of immobility was measured at the last 4 mins. A mouse was judged immobile when it ceased all active behaviors (i.e. struggling, swimming, and jumping) and remained passively floating or making minimal movements necessary to maintain the nostrils above water (3, 18). A high percentage of time floating was interpreted as an indication of depressive-like response. The animals were placed into the testing apparatus under illumination (60 lux). All the behaviors were recorded using EthoVision^®^ XT 8.

### Novel object test

The object location task (OLT) and the novel object recognition task (NORT) are two effective behavioral tasks to assess cognitive functions. These tests exploit the inherent preference of mice for the novelty to reveal memory for previously encountered objects. While OLT primarily evaluates spatial learning, which relies on hippocampal activity, NORT evaluates non-spatial learning of object identity, which relies on multiple brain regions (20). The mice were placed in an open field box with two familiar objects for 5 min. In this test, the light should not be too bright. For the NORT, mice were left to explore freely the objects and the box. After one hour, one of the objects was replaced with a new object, and the mice were left to explore again and distinguish between the familiar and novel object. For the OLT, on 2^nd^ day, mice explored the same familiar object and after one hour the location was changed to a new spot. All the behaviors were recorded using EthoVision^®^ XT 8.

### Cells isolation

Mice were euthanized using halothane followed by cardiac puncture to confirm death. Animals were then thoroughly perfused with ice-cold PBS, pH 7.4, for 5 mins. Heads were removed and skulls were stripped of all flesh. Surgical scissors were used to remove skull tops in a clockwise manner. The skulls were immediately placed in ice-cold RPMI media. Meninges were carefully removed from the interior aspect of skulls and surfaces of brains with forceps (21, 22). Hippocampus was separated from the brain parenchyma using surgical forceps. Single cell suspension from meninges and hippocampus (HPC) was prepared by enzymatic digestion in RPMI containing 220 U/mg Collagenase IV (Gibco, Waltham, Massachusetts), 13 U/mg DNase I (Sigma, St. Louis, Missouri), and 5% iFCS (inactivated fetal calf serum) (Gibco) (digestion buffer) for 30 min at 37°C under constant rotation. The resulting suspension was mechanically squeezed through a 100 µM sterile cell strainer (Falcon) followed by centrifugation at 1200 rpm for 10 min at 4°C. Supernatant was discarded and the cells were resuspended in RPMI medium and checked for viability and cell number by trypan blue staining.

### Flow cytometry

Antibodies used for flow cytometry analysis were as follows: CD3ϵ, CD4, IL-4, IL-5, IL-6, IL-10, IL-13, CD45, CD11b, F4/80, and MHC II purchased from BD Biosciences (Franklin Lakes, New Jersey) and eBioscience (San Diego, California). For staining of cell surface markers, cells (1 x 10^6^) were labelled and washed in PBS containing 1% BSA (Roche, Switzerland) and 0.1% NaN_3_ (FACS buffer). For detection of intracellular cytokines, cells were seeded at a density of 2 x 10^6^ cells/well in a complete RPMI culture medium and stimulated with 50 ng/ml phorbol myristate acetate (PMA), 250 ng/ml ionomycin, and 200 µM monensin (all from Sigma) for 6 hr at 37°C in a humidified atmosphere containing 5% CO_2_. After the incubation period, cells were harvested, washed, fixed in 2% (w/v) paraformaldehyde, permeabilized with 0.5% saponin buffer, and then stained for cytokine production as previously described (23, 24). Fluorescence minus one (FMO) was used as a control for intracellular staining whereby all fluorophores were added except one, i.e. cytokine fluorophore, to detect positive signal. Acquisition was performed using BD LSRFortessa (BD Biosciences) and data were analyzed using FlowJo software (Treestar, Ashland, Oregon).

### Tissue homogenate for cytokine analysis

Brain parenchyma (hippocampus) were collected and homogenized in RIPA buffer containing 1% protease inhibitor cocktail (Sigma-Aldrich, St. Louis, MO, US, catalogue no. P8340). The homogenate was spun down and the supernatant was isolated for cytokine analyses (24). Cytokines (IL-1β, IL-4, IL-5, IL-6, IL-10, IL-13, IL-17, IFNγ, and TNFα all from BD Pharmingen) were measured in the protein extracts by sandwich ELISA as described previously (23, 24). Cytokine values were normalized according to the protein content measured by Pierce BCA Protein Assay Kit (Thermo Fisher Scientific, catalogue no. 23225).

### Statistics

Statistical analysis was conducted using GraphPad Prism 7 software (http://www.prism-software.com). Data were calculated as mean ± SD. Statistical significance was determined using the unpaired Student’s *t*-test defining differences to uninfected mice as significant (*, *P* ≤ 0.05; **, *P* ≤ 0.01; ***, *P* ≤ 0.001).

## Results

### Immune cell profile in the meninges and brain parenchyma of C57BL/6 sub-strains under steady state condition

**Figure 1:**
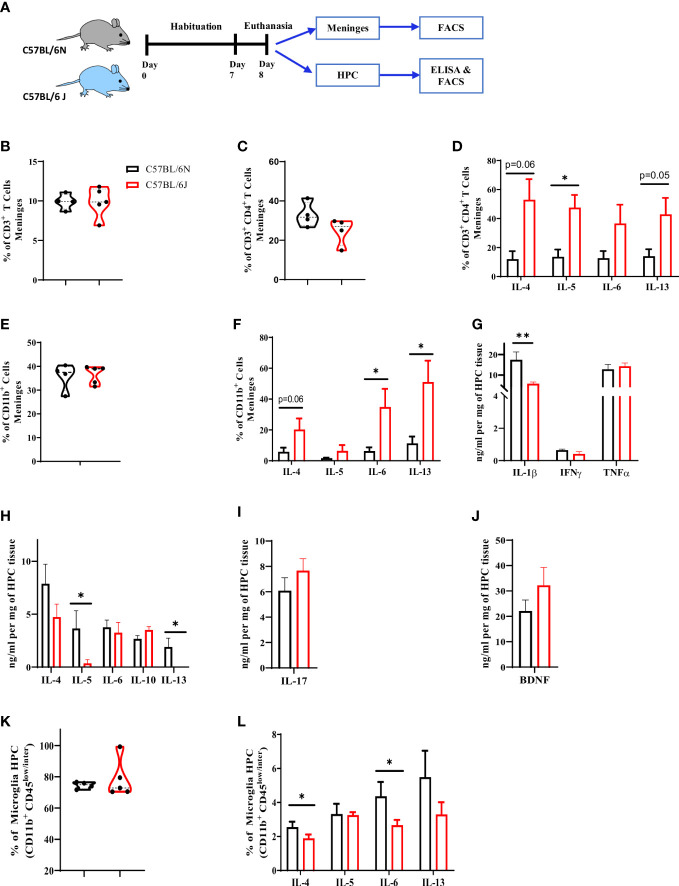
Characterization of immune cell profile in meninges and hippocampus in C57BL/6 sub-strains. **(A)** Experimental design. Meninges and HPC were collected from naïve adult mice and the proportion of immune cells and level of cytokines were assessed using flow cytometry and ELISA. **(B)** Frequency of CD3^+^ T cells, **(C)** CD4^+^ T cells, and **(D)** IL-4, IL-5, IL-6 and IL-13-expressing CD4^+^ T cell in meninges. **(E)** Frequency of CD11b+ cells and **(F)** IL-6, IL-4, IL-5, and IL-13-expressing CD11b^+^ cell in meninges. The level of **(G)** Type 1, **(H)** Type 2, and **(I)** Type 17 cytokines as well as **(J)** BDNF using ELISA and normalized to mg of HPC tissue. **(K)** Frequency of microglia (CD11b^+^ CD45^low/inter^) in HPC. **(L)** frequency of IL-6, IL-4, IL-5, and IL-13-producing microglia under steady state. Results are representative of three independent experiments with 4–7 mice/group. Data are expressed as mean ± S.E.M. * P < 0.05, ** P < 0.001 by two-tailed unpaired Student t-test.

We first investigated whether immune cell proportion and cytokine production in meninges and brain parenchyma would be similar between C57BL/6 sub-strains. To address that, meninges and brain parenchyma, particularly hippocampus (HPC), were collected and the immune profile was characterized using flow cytometry and ELISA (**Figure 1A**). Characterization of the T cell population in meninges indicated a comparable frequency of CD3^+^ (**Figures 1B**, **S1A**) and CD3^+^ CD4^+^ T cell populations (**Figure 1C**) in the two sub-strains. CD3^+^ CD4^+^ T cell population in B6J mice were, however; producing more IL-4, IL-5, IL-6, and IL-13 (**Figure 1D**), suggesting a higher tendency of T cells in B6J mice to produce more type 2 cytokines when compared to their counterparts in B6N mice. We then assessed changes in the CD11b^+^ population in meninges (**Figure S1B**). Under steady state, B6N and B6J mice had a comparable frequency of the CD11b^+^ population (**Figure 1E**). Similar to T cells, the basal level of type 2 cytokine production by CD11b^+^ population in B6J seemed to be higher compared to B6N mice (**Figure 1F**). Indeed, CD11b^+^ population in B6J was producing more IL-4, IL-5, IL-6, and IL-13 (**Figure 1F**). Together, these data indicated whereas the frequency of T and CD11b^+^ populations was comparable in the meninges of C57BL/6 sub-strains, the basal ability of these cells to produce type 2 cytokines was different.

Next, we sought to characterize the cytokine pattern in HPC in the two sub-strains. For type 1 cytokines, B6J displayed a significant reduction in IL-1β and a comparable level of IFNγ and TNFα (**Figure 1G**). For type 2 cytokines, B6J mice exhibited a significant reduction in IL-5 and IL-13 while maintaining a similar level of IL-4 and IL-6 (**Figure 1H**). Furthermore, we did not note any major differences in either the regulatory cytokine IL-10 (**Figure 1H**), IL-17 production (**Figure 6I**) or BDNF (**Figure 1J**) in the two sub-strains. We then analysed the profile of microglia population, which is the brain resident macrophages, using flow cytometry (**Figure S1C**). C57BL/6 sub-strains had a comparable microglia frequency (**Figure 1K**). Interestingly, however; these microglia were different in their cytokine production ability. Microglia in B6J mice produced less IL-4 and IL-6 when compared to B6N (**Figure 1L**). Thus, under steady state and unlike meninges, microglia in B6J mice produced less type 2 cytokines compared to B6N.

### Affective and cognitive behavior in C57BL/6 sub-strains

**Figure 2:**
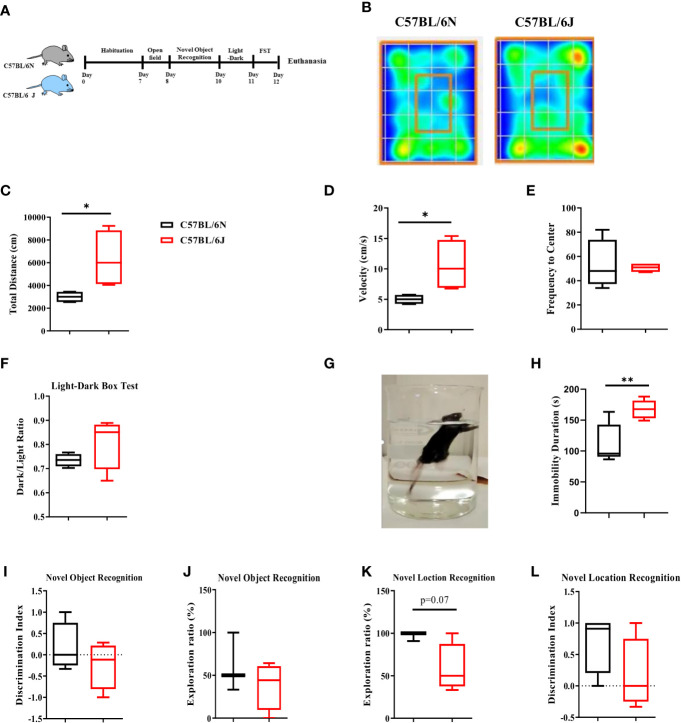
Affective and cognitive behavior in C57BL/6 sub-strains. **(A)** Experimental design set 1. Naïve adult C57BL/6N and C57BL/6J mice were assessed for affective and cognitive behavior using a battery of tests that started with the least to the most stressful ones. **(B)** Heat map of the sub-strains movement pattern in open field test under steady state. **(C)** Total distance travelled, **(D)** Velocity, and **(E)** frequency to the centre were measured. **(F)** Dark-to-light transition ratio using light-dark box test. **(G)** Depression-like behavior was assessed using FST **(H)** and total immobility time was determined using EthoVision® XT 8. **(I)** Exploration ratio and **(J)** Discrimination index in NOR test. **(K)** Exploration ratio and **(L)** Discrimination index in OLT test. Results are representative of two independent experiments with 4–7 mice/group. Data are expressed as mean ± S.E.M. *P < 0.05, **P < 0.001 by two-tailed unpaired Student t-test.

We then questioned whether the alteration in immune cell profile in meninges and HPC would be associated with distinct patterns of affective and cognitive behavior. To address that, B6J and B6N adult mice were habituated for 7 days and then exposed to a battery of behavioral tests, starting with the least to the most stressful ones (**Figure 2A**). Open field and light-dark box tests were used to assess basal locomotor activity and anxiety-like behavior. Our analyses indicated a distinct movement pattern of C57BL/6 sub-strains (**Figure 2B**) in the open field test. Furthermore, B6J mice travelled a longer distanced (**Figure 2C**) and had a higher level of velocity (**Figure 2D**) when compared to B6N mice. Together, these data suggested a distinct locomotor activity of the two sub-strains. For anxiety-like behavior, there were no differences in the frequency to the centre, using open field test, (**Figure 2E**) or dark-to-light transition ratio in the light-dark box test (**Figure 2F**). Together, these data suggested a comparable level of anxiety-like behavior under steady state between the two sub-strains.

We then assessed the immobility pattern as an indicator of depression-like behavior using Forced Swim Test (FST) (**Figure 2G**). Under steady state, B6J mice displayed longer immobility duration compared to the B6N substrain (**Figure 2H**), suggesting that B6J mice might have a higher propensity to develop depression-like behavior.

We next evaluated the cognitive functions using Novel Object Recognition Test (NORT) and Novel Object Location Test (OLT). NORT is a hippocampal-independent non-spatial learning and memory whereby mice were allowed to explore two identical objects, one of these objects were then replaced with a novel one (20). Under steady state, our analyses indicated a trend of lower exploration time (**Figure 2I**) and discrimination index (**Figure 2J**) between B6N and B6J. This trend was, however; not up to the significance level. Next, we assessed the hippocampus-dependent spatial learning and memory using OLT whereby mice were allowed to explore two identical objects, one of these objects was then placed in a different location. We noted a trend of lower exploration ratio (**Figure 2K**) with a similar discrimination index (**Figure 2L**) in B6J mice compared to B6N. Together, these data suggested a modest alteration in the basal level of hippocampus-dependent and independent spatial learning and memory in B6J mice compared with B6N.

### Microbial and environmental factors have an influential impact on the locomotor activity of C57BL/6 sub-strains

**Figure 3:**
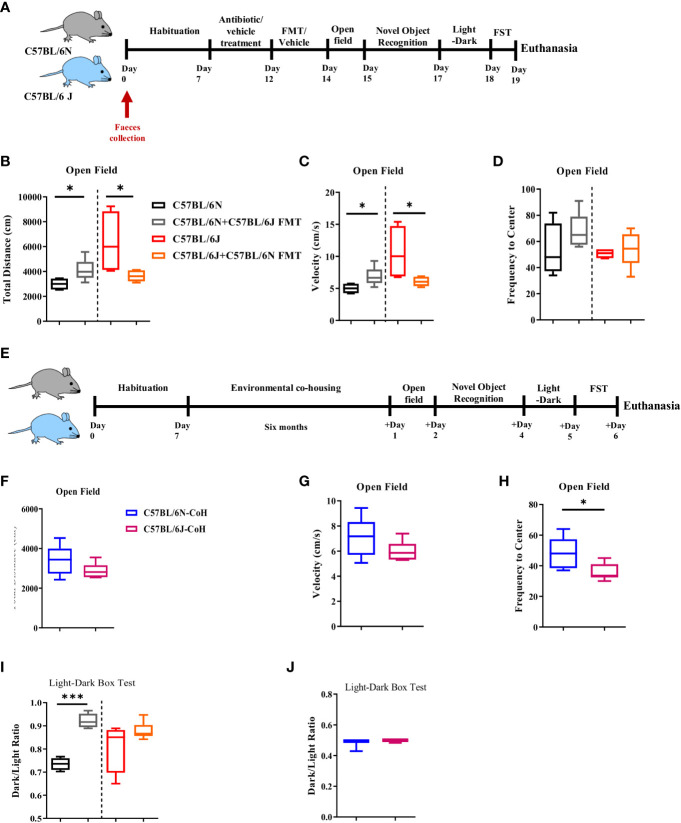
Microbial and environmental factors influence locomotor activity of C57BL/6 sub-strains. **(A)** Experimental design set 2. Faeces were collected from C57BL/6N and C57BL/6J mice day 0. These mice were treated for 5 days with antibiotic cocktail for microbiome clearance. Faecal microbiota transfer (FMT) was then done through oral gavage. **(B)** Total distance travelled, **(C)** Velocity, and **(D)** Frequency to the centre in C57BL/6N and C57BL6/J upon FMT are depicted. **(E)** Experimental design set 3. In the third setup, C57BL/6N and C57BL/6J mice were environmentally co-housed for six months followed by an assessment of the affective and cognitive behaviors. **(F)** Total distance travelled, **(G)** Velocity, and **(H)** Frequency to the centre after long-term co-housing are depicted. **(I)** Dark-to-light ratio using light-dark box test was measured upon FMT, and **(J)** after long-term co-housing. Results are representative of two independent experiments with 5–7 mice/group. Data are expressed as mean ± S.E.M. *P < 0.05, ***P < 0.0001 by two-tailed unpaired Student t-test.

Given that our findings of affective and cognitive behaviors are in accordance with previously published reports and contrast with others (2–4, 9–11), we sought to disentangle the contribution of the microbial and environmental factors to the phenotype noted. To examine the role of the microbiome, we treated B6J and B6N with an antibiotic cocktail for five consecutive days followed by reciprocal faecal microbiota transfer (FMT), i.e., B6J received FMT from B6N and vice versa. The mice were then exposed to affective and cognitive behavioral tests (**Figure 3A**). In the open field test, we noted a significant increase in the distance travelled (**Figure 3B**) and velocity (**Figure 3C**) of B6N mice after receiving FMT from B6J mice. In the same manner, B6J mice receiving FMT of B6N displayed a reduction in the total distance travelled (**Figure 3B**) and velocity (**Figure 3C**) when compared to its naïve counterpart, B6J. No difference was noted in the frequency of centre visits before or after FMT in B6J or B6N mice (**Figure 3D**). Together, these data suggested a role of gut microbiome in locomotor activity as FMT recipient mice displayed a locomotor activity similar to the donor. We next assessed the role of environmental factors whereby mice were environmentally co-housed for 6 months and then exposed to affective and cognitive behavioral tests (**Figure 3E**). In the open field test, the difference in the total distance travelled (**Figure 3F**) and velocity (**Figure 3G**) between B6N and B6J mice, previously demonstrated, vanished after the period of environmental co-housing. Together, these data further supported the influential role of environmental and microbial factors in locomotor activity in C57BL/6 sub-strains. Of note, the frequency to the centre was significantly reduced in the co-housed B6J mice compared to the co-housed B6N, suggesting a higher propensity of B6J mice to develop a higher level of anxiety-like behavior after long-term co-housing (**Figure 3H**). Using the light-dark box test, we noted similar dark-to-light transition ratio after FMT (**Figure 3I**) and long-term environmental co-housing (**Figure 3J**) in B6J and B6N mice, suggesting a similar level of anxiety-like behavior in the two sub-strains.

### Immobility pattern in C57BL/6 sub-strains is more resilient to microbial and environmental factors

**Figure 4:**
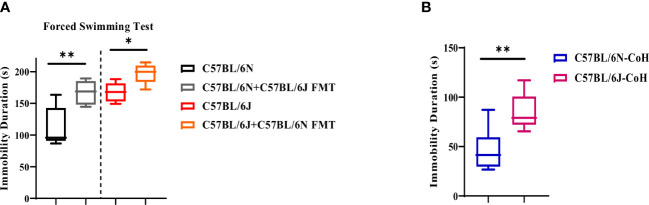
Microbial and environmental factors are dispensable for depression-like behavior pattern in C57BL/6 sub-strains. Immobility duration in C57BL/6N and C57BL6/J mice upon **(A)** FMT and **(B)** after long-term co-housing. Results are representative of three independent experiments with 5–7 mice/group. Data are expressed as mean ± S.E.M. * P < 0.05, ** P < 0.001, by two-tailed unpaired Student t-test.

Next, we investigated the involvement of gut microbiome and environmental factors in the development of depression-like behavior in the two sub-strains (**Figure 2H**). Under FMT condition, we noted a marginal role of microbiota in immobility pattern. Even though FMT promoted the immobility duration in both strains, the distinctive pattern of immobility duration, i.e., higher immobility time in B6J compared to B6N, was maintained in the two sub-strains before and after FMT (**Figure 4A**). Similarly, under long-term environmental co-housing, B6J mice displayed higher immobility duration (**Figure 4B**), compared to B6N. Together, our data demonstrated a dispensable role for microbial and environmental factors in immobility pattern in these sub-strains.

### Impact of microbial and environmental factors on cognitive function

In our characterization, we noted modest alteration in cognitive functions between the two sub-strains, and thus we sought to investigate whether changes in microbial and environmental factors may change the cognitive behavior pattern. For hippocampal-independent non-spatial learning and memory, and similar to NORT steady state condition, B6N and B6J displayed similar exploration ratio (**Figure S2A**). Of interest, the discrimination index in B6N FMT recipient mice was reduced to a level comparable to B6J ones (**Figure S2B**). Similarly, the discrimination index in B6J mice receiving FMT from B6N was enhanced to a level comparable to B6N mice (**Figure S2B**). After long-term environmental co-housing, the two sub-strains had similar exploration ratio (**Figure S2C**) and discrimination index (**Figure S2D**). However, B6J had a significant reduction in the total exploration time (**Figure S2E**). Assessment of hippocampal-dependent spatial learning and memory using OLT indicated that FMT transfer from B6J to B6N led to a reduction in the exploration ratio (**Figure S2F**) and discrimination index (**Figure S2G**), reproducing a pattern similar to B6J mice. In contrast, FMT transfer from B6N to B6J did not cause major alteration in either the exploration ratio (**Figure S2F**) or discrimination index (**Figure S2G**). After long-term co-housing, B6N and B6J mice exhibited a comparable exploration ratio (**Figure S2H**) and discrimination index pattern (**Figure S2I**). Together, these data indicated a differential impact of microbial and environmental factors on HPC-dependent and independent learning and memory.

### Marginal impact of microbiota and environmental factors on immune cell populations in the meninges

**Figure 5:**
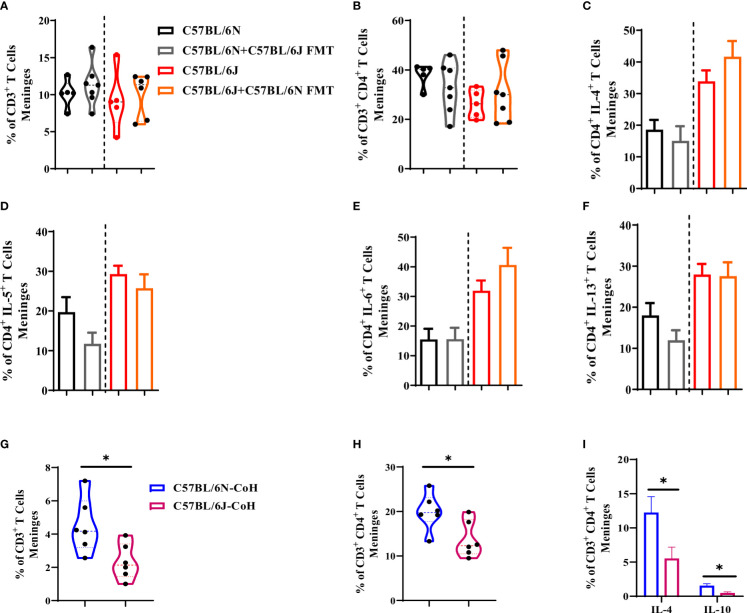
meningeal T cells are resilient to changes in microbial and environmental factors. **(A)** Frequency of CD3^+^ T cells, **(B)** CD4^+^ T cells, and **(C)** IL-4, **(D)** IL-5, **(E)** IL-6, and **(F)** IL-13-expressing CD4^+^ T cell in C57BL/6N and C57BL/6J upon FMT. **(G)** Frequency of CD3^+^ T cells, **(H)** CD4^+^ T cells, and **(I)** IL-4 and IL-10-expressing CD4^+^ T cell in C57BL/6 sub-strains after long-term environmental co-housing. Results are representative of three independent experiments with 5–7 mice/group. Data are expressed as mean ± S.E.M. *P < 0.05 by two-tailed unpaired Student t-test.

We then analysed how microbiota and environmental factors influenced the immune cell profile in brain. Characterization of T cells after FMT in meninges indicated a comparable frequency of CD3^+^ (**Figure 5A**) and CD3^+^ CD4^+^ T cell populations (**Figure 5B**) in the two sub-strains, similar to the findings under steady state.

Notably, the cytokine production ability by CD3^+^ CD4^+^ T cell population was not altered in response to FMT (**Figures 5C–F**) when compared to naïve mice. For instance, B6N produced a comparable amount of IL-4 (**Figure 5C**), IL-5 (**Figure 5D**), IL-6 (**Figure 5E**), and IL-13 (**Figure 5F**) before and after FMT. The same thing was also noted for B6J mice (**Figures 5C–F**). Together, these data suggested that gut microbiota alteration did not have a major impact on T cell profile in meninges in C57BL/6 sub-strains. Under long term environmental co-housing, we noted a significant reduction in CD3^+^ (**Figure 5G**) and CD3^+^ CD4^+^ T cell populations (**Figure 5H**) in B6J mice compared to B6N mice. Of note, T cell production of IL-4 and IL-10 was also reduced in B6J mice (**Figure 5I**).

Similar impact of microbial and environmental factors was also noted on CD11b^+^ population in the meninges. In fact, under gut microbiome transfer condition, the frequency of CD11b^+^ population (**Figure S3A**) was comparable between the two sub-strains. The level of IL-4 (**Figure S3B**), IL-5 (**Figure S3C**), IL-6 (**Figure S3D**), and IL-13 (**Figure S3E**) were comparable before and after FMT in each sub-strain, thus maintaining the same profile of higher tendency of type 2 cytokine production by CD11b^+^ population in B6J mice. Under environmental co-housing condition, we found a significant reduction in CD11b^+^ population in B6J when compared to B6N mice (**Figure S3F**). Except for IL-4 which was significantly reduced in B6J mice (**Figure S3G**), CD11b^+^ cells produced a comparable level of IL-6 and IL-10 in the two sub-strains (**Figure 3G**). Together, this data suggested that long-term co-housing resulted in reduction in meninges immune cell frequency and their type 2 cytokine production.

### Microglia in HPC are responsive to changes in microbial and environmental factors

**Figure 6:**
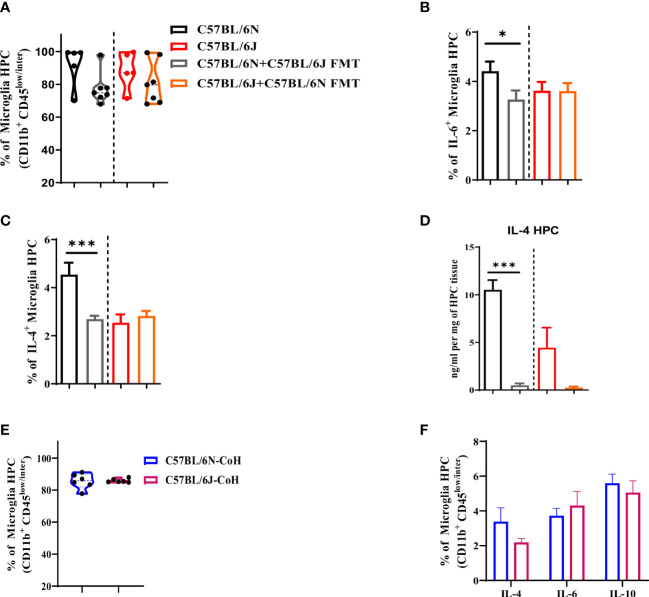
Microglia are responsive to microbial and environmental factors in C57BL/6 sub-strains. Hippocampus were collected from C57BL/6N and C57BL/6J under different experimental conditions to assess changes in microglia proportion and their cytokine production. **(A)** Frequency of microglia (CD11b^+^ CD45^low/inter^). Frequency of microglia (CD11b^+^ CD45^low/inter^) producing **(B)** IL-6 and **(C)** IL-4 upon FMT. **(D)** IL-4 level measured in HPC upon FMT using ELISA and normalized to mg of HPC tissue. **(E)** Frequency of microglia (CD11b^+^ CD45^low/inter^) and **(F)** frequency of IL-4, IL-6, and IL-10-producing microglia in C57BL/6N and C57BL/6J after long-term co-housing. Results are representative of three independent experiments with 5–7 mice/group. Data are expressed as mean ± S.E.M. * P < 0.05, *** P < 0.0001 by two-tailed unpaired Student t-test.

Microglia play key roles in cognitive and affective behavior. Furthermore, microglia are highly sensitive to changes in gut microbiota and environmental factors (19, 25–28). Thus, we assessed microglia response to FMT and long-term co-housing in HPC. Under FMT condition, gut microbiota transfer did not alter microglia frequency in either B6J or B6N (**Figure 6A**). Of interest, the B6N FMT recipient mice displayed a significant reduction in microglia production of IL-6 (**Figure 6B**) and IL-4 (**Figure 6C**). Notably, these cytokines were reduced to the level noted in B6J mice. In support, the total IL-4 in HPC was significantly reduced in B6N mice after FMT (**Figure 6D**). In contrast, transfer of B6N’s gut microbiome to B6J did not cause significant alteration in microglia production of either IL-6 (**Figure 6B**) or IL-4 (**Figure 6C**) as well as the total level of IL-4 (**Figure 6D**). Together, these data indicated that after short-term antibiotic treatment and FMT, there was a unidirectional impact of gut microbiota in altering cytokine production ability of microglia in HPC. Under long-term co-housing condition, there was a comparable frequency of microglia in B6J and B6N (**Figure 6E**). Of note, microglia produced similar amount of type 2 cytokines (IL-4, IL-6, and IL-10) (**Figure 6F**). The long-term co-housing might have helped in normalizing the microbiome differences between the two strains. Collectively, these data suggested an influential impact of microbial and environmental factors on microglia cytokine production ability.

## Discussion

C57BL/6 mice are one of the most widely used inbred wildtype and genetically engineered mouse model in biomedical sciences (2, 11, 29). This strain has been generated decades ago and several sub-strains were then subsequently driven. Studies have indicated variations in several phenotypic traits and behavior between the different sub-strains (2–4, 9–11). While some of the behavioral variations could be genetically driven, others are not consistent in the literature (3, 9, 10, 29), suggesting the involvement of other factors like environmental and/or microbial factors. In the present study, we characterized cognitive and affective behavior in B6J and B6N in association with the immune cell profile in brain. We further demonstrated the contribution of genetic, microbial and environmental factors to cognitive and affective behaviors in C57BL/6 sub-strains. Our analyses demonstrated a distinct pattern of immune cell profile in the two sub-strains whereby a higher basal level of type 2 cytokine by T cells and CD11b^+^ myeloid cells in the meninges along with reduction in type 2 cytokines, i.e., IL-4, production by microglia in HPC, was noted in B6J compared to B6N sub-strain. This immune profile was associated with changes in behavior like locomotor activity, immobility pattern and spatial and non-spatial learning and memory abilities between B6J and B6N under steady state. Some of the noted differences between the two sub-strains were most likely driven by microbial and environmental factors, while others were genetically driven. In fact, our data demonstrated that locomotor activity was highly sensitive to alteration in microbial and environmental factors as gut microbiota transfer and co-housing led to a remarkable change in the locomotor activity. In contrast, high-immobility time in FST, as an indicative of depression-like behavior, was highly resilient, as immobility pattern in the two sub-strains was the same under all the tested conditions. We further noted a differential influence of microbiome and environmental factors on spatial and non-spatial learning and memory abilities. Of interest, changes in the phenotypic behavior in response to environmental and microbial factors were associated with changes in immune cells profile. While microglia were highly sensitive to alteration in gut microbiome, immune cells in meninges were more resilient. Collectively, our findings demonstrated that alteration in environmental factor impacts gut microbiome which alter immune cell profile in brain parenchyma and subsequently cognitive and affective behavior. Our findings demonstrated the affective and cognitive domains which were sensitive to changes in microbial and environmental factors and further shed the light on the importance of characterizing the available sub-strains to select the one that fits best for the study purpose.

Recent studies have indicated that phenotype development is dependent on host genes, microbial factors, and/or the interaction between them. Therefore, the interplay between host genetics, environmental factors and microbiome composition has a great influence on phenotype development (13). In the present study, we sought to disentangle the contribution of host genes, microbiome and environmental factors to the phenotypic variation in C57BL/6 sub-strains. We first analysed the locomotor activity in the two sub-strains and noted a distinct pattern of locomotor activity in B6J compared to B6N mice. B6J displayed a higher velocity that was associated with increase in the total distance moved. The difference in locomotor activity was previously reported. While the findings of some studies are in accordance with ours (3, 10, 11), others have indicated the opposite whereby B6N mice displayed higher activity (3). These contrasting findings suggested the contribution of factors other than host genes, like for instance microbial and/or environmental factors. Faecal microbiome transfer was used to assess the role of microbiome, whereases long-term co-housing helped in assessing the impact of both environmental factors and microbiome, as mice are coprophagic. Gut microbiome transfer from B6J to B6N led to increase in the velocity and the total distance in the recipient B6N mice compared to their naïve counterparts, and vice versa in the case of B6J FMT recipient mice. Alteration in gut microbiome could be likely driven by either genetic or environmental factors. The latter is most likely the case in the present study, as mice co-housed for long-time displayed a comparable velocity and total distance (locomotor activity). Thus, these findings suggested that variations in environmental factors resulted in alteration in gut microbial composition that led to differences in locomotor activity. These results are in accordance with a previously published report that indicated a role of gut microbiota in modulating locomotor activity through altering neuronal function in *Drosophila* (30).

Unlike locomotor activity profile, the pattern of high-immobility time in FST, as an indicator of depression-like behavior, seems to be consistent under all tested conditions. Under steady state, the analyses of FST indicated an increase in immobility time in B6J mice, suggesting a higher basal level of depression-like behavior in B6J compared to B6N mice. The mentioned immobility pattern of the two sub-strains was reproducible, and even more robust, under FMT and long-term environmental co-housing conditions. Thus, increased immobility time in B6J mice could be likely driven by genetic factors as it was more resilient to changes in environmental and microbial factors. The higher basal level of immobility time may indicate a higher propensity for depression-like behavior development in B6J mice which might make this model not the best choice for testing this phenotype but this has to be further validated in a depression-induced experimental model. Of note, opposite depression-like behavior pattern was reported elsewhere, suggesting laboratory-specific variations that develop over time and highlighting the importance of characterizing the available laboratory sub-strains to select the best model that fits best for the study purpose (10).

Notably, there was no difference in anxiety-like behavior between the two sub-strains as they displayed similar frequency to the centre in the open field test and had a comparable level of dark-to-light transition ratio which is in agreement with previously published report (3). Thus, the two sub-strains had a similar basal level of anxiety-like behavior. Of note, B6J mice under FMT and co-housing conditions displayed a trend of a lower frequency to the centre relative to its B6N counterparts, suggesting a higher tendency of B6J to develop anxiety-like behavior under less favourable conditions which may explain why in some studies B6J displayed higher anxiety level compared with B6N (29).

In addition to affective behavior, previous report also assessed cognitive behavior whereby they could not detect a significant difference in spatial learning and memory capacity (11, 29). Our data, however; demonstrated a modest alteration in hippocampus-dependent and independent learning and memory in the two sub-strains. In hippocampus-independent non-spatial learning and memory, B6J mice displayed a trend of lower exploration ratio and discrimination index. This pattern was replicated after gut microbiome transfer as recipient mice displayed a similar discrimination index as the donor mice. For instance, B6N mice receiving FMT from B6J mice displayed a discrimination index similar to that of naïve B6J mice, and vice versa, suggesting a bidirectional impact of gut microbiota on hippocampus-independent non-spatial learning and memory. In contrast, gut microbiome impact on the pattern of hippocampus-dependent spatial learning and memory seemed to be unidirectional because B6N mice receiving FMT from B6J had a reduction in total exploration time and discrimination index to a level comparable to B6J mice but not vice versa. This could be explained by our findings on microglia profile in HPC whereby the impact of gut microbial factors on microglia was unidirectional. In fact, gut microbiota transfer from B6J to B6N did significantly reduce the expression of IL-4 and IL-6 by microglia to a level comparable to the ones recorded in B6J mice, but not vice versa, which might have driven the relative reduction in discrimination index and exploration ration in OLT test. The unidirectional phenomenon can be explained by the fact that reciprocal FMT protocol can help in supplementing a relatively sparse microbial population with taxa from a richer source, but it may not be efficient in repopulating the mice with a less rich and diverse microbial populations than was originally exist (15). Depleting a rich microbial population may require a longer time than supplementing. Our data can support this hypothesis as long-term environmental co-housing did normalize many variations between the co-housed mice. For instance, co-housed mice displayed a comparable level of cytokine production by microglia in HPC which was associated with a similar exploration ratio and discrimination index in hippocampus-dependent and independent learning and memory. A control group with a shorter time of co-housing might have been beneficial to control for age as a possible confounding factor, but it may not have been ideal for enriching sparse microbial populations. Collectively, our analyses indicated a differential impact of gut microbiota on hippocampus-dependent and independent learning and memory. It further highlighted the sensitivity of microglia in HPC to alterations in gut microbiota and environmental factors, most likely through the gut-brain axis (19, 25–28), which subsequently impact specific cognitive domains.

Unlike microglia, immune cells in meninges were more resilient to changes in gut microbiome and environmental factors. Under steady state, our analyses indicated that while T cells and CD11b^+^ myeloid cells frequency in meninges were comparable between the sub-strains, their basal level of type 2 cytokines production was remarkably different. In B6J mice, meningeal T and CD11b cells produced more type 2 cytokines: namely IL-4, IL-5, IL-6, and IL-13 under steady state. This pattern of cytokine production was reproducible under FMT condition, suggesting a higher resilience of meningeal immune cells to gut microbiota alteration. Long-term co-housing did, however; lead to a reduction in type 2 cytokines production in B6J mice which was associated with enhanced production of type 2 cytokines by microglia in B6J HPC to a level comparable to the one noted in B6N mice after co-housing. Thus, type 2 cytokine pattern noted under steady state and long-term cohousing suggests a dynamic cytokine balance between meninges and brain parenchyma.

Overall, our results demonstrated an association between changes in gut microbiome, environmental factors, brain immune cells profile and manifestation of distinct patterns of cognitive and affective behavior. We also indicated that while microglia in HPC were the most responsive brain resident immune cells to alterations in gut microbiome, immune cells in meninges were more resilient. Our data further suggested a dynamic balance of cytokine production by immune cells in meninges and brain parenchyma. The current study also emphasized the differences between the sub-strains pointing towards the importance of characterizing the laboratory available strains to select the most appropriate one that fits best for the study purpose.

## Data availability statement

The original contributions presented in the study are included in the article/**Supplementary Material**. Further inquiries can be directed to the corresponding authors.

## Ethics statement

The animal study was reviewed and approved by the Animal Research Ethics Committee of the Faculty of Health Sciences, University of Cape Town.

## Author contributions

Conceptualization, NA and IB. Methodology, NA, IB, PM, and TB. Investigation, NA, IB, PM, and TB. Writing – Original Draft, NA. Writing – Review & Editing, NA, IB, PM, and FB. Supervision, NA and FB. Project Administration, NA and IB, Resources, FB. Funding Acquisition, FB. All authors contributed to the article and approved the submitted version.

## References

[1] Mekada K Abe K Murakami A Nakamura S Nakata H Moriwaki K. Genetic differences among C57BL/6 substrains. Exp Anim (2009) 58:141–9. doi: 10.1538/expanim.58.141 19448337

[2] Mekada K Yoshiki A. Substrains matter in phenotyping of C57BL/6 mice. Exp Anim (2021) 70:145–60. doi: 10.1538/expanim.20-0158 PMC8150240 33441510

[3] Simon MM Greenaway S White JK Fuchs H Gailus-Durner V Wells S. A comparative phenotypic and genomic analysis of C57BL/6J and C57BL/6N mouse strains. Genome Biol (2013) 14:R82. doi: 10.1186/gb-2013-14-7-r82 23902802 PMC4053787

[4] Keane TM Goodstadt L Danecek P White MA Wong K Yalcin B. Mouse genomic variation and its effect on phenotypes and gene regulation. Nature (2011) 477:289–94. doi: 10.1038/nature10413 PMC3276836 21921910

[5] Mattapallil MJ Wawrousek EF Chan CC Zhao H Roychoudhury J Ferguson TA. The Rd8 mutation of the Crb1 gene is present in vendor lines of C57BL/6N mice and embryonic stem cells, and confounds ocular induced mutant phenotypes. Invest Ophthalmol Vis Sci (2012) 53:2921–7. doi: 10.1167/iovs.12-9662 PMC3376073 22447858

[6] Kang SK Hawkins NA Kearney JA. C57BL/6J and C57BL/6N substrains differentially influence phenotype severity in the Scn1a (+/-) mouse model of dravet syndrome. Epilepsia Open (2019) 4:164–9. doi: 10.1002/epi4.12287 PMC6398090 30868126

[7] Akinola LS McKiver B Toma W Zhu AZX Tyndale RF Kumar V. C57BL/6 substrain differences in pharmacological effects after acute and repeated nicotine administration. Brain Sci (2019) 9:244. doi: 10.3390/brainsci9100244 31546627 PMC6827359

[8] Freeman HC Hugill A Dear NT Ashcroft FM Cox RD. Deletion of nicotinamide nucleotide transhydrogenase: a new quantitive trait locus accounting for glucose intolerance in C57BL/6J mice. Diabetes (2006) 55:2153–6. doi: 10.2337/db06-0358 16804088

[9] Bryant CD Zhang NN Sokoloff G Fanselow MS Ennes HS Palmer AA. Behavioral differences among C57BL/6 substrains: implications for transgenic and knockout studies. J Neurogenet (2008) 22:315–31. doi: 10.1080/01677060802357388 PMC3697827 19085272

[10] Matsuo N Takao K Nakanishi K Yamasaki N Tanda K Miyakawa T. Behavioral profiles of three C57BL/6 substrains. Front Behav Neurosci (2010) 4:29. doi: 10.3389/fnbeh.2010.00029 20676234 PMC2912075

[11] Ashworth A Bardgett ME Fowler J Garber H Griffith M Curran CP. Comparison of neurological function in males and females from two substrains of C57BL/6 mice. Toxics (2015) 3:1–17. doi: 10.3390/toxics3010001 27081652 PMC4829364

[12] Baker M. 1,500 scientists lift the lid on reproducibility. Nature (2016) 533:452–4. doi: 10.1038/533452a 27225100

[13] Stappenbeck TS Virgin HW. Accounting for reciprocal host-microbiome interactions in experimental science. Nature (2016) 534:191–9. doi: 10.1038/nature18285 27279212

[14] Kafkafi N Agassi J Chesler EJ Crabbe JC Crusio WE Eilam D. Reproducibility and replicability of rodent phenotyping in preclinical studies. Neurosci Biobehav Rev (2018) 87:218–32. doi: 10.1016/j.neubiorev.2018.01.003 PMC6071910 29357292

[15] Ericsson AC Personett AR Turner G Dorfmeyer RA Franklin CL. Variable colonization after reciprocal fecal microbiota transfer between mice with low and high richness microbiota. Front Microbiol (2017) 8:196. doi: 10.3389/fmicb.2017.00196 28280484 PMC5322181

[16] Surana NK Kasper DL. Moving beyond microbiome-wide associations to causal microbe identification. Nature (2017) 552:244–7. doi: 10.1038/nature25019 PMC5730484 29211710

[17] Velazquez EM Nguyen H Heasley KT Saechao CH Gil LM Rogers AWL. Endogenous enterobacteriaceae underlie variation in susceptibility to salmonella infection. Nat Microbiol (2019) 4:1057–64. doi: 10.1038/s41564-019-0407-8 PMC6533147 30911125

[18] Boehme M van de Wouw M Bastiaanssen TFS Olavarria-Ramirez L Lyons K Fouhy F. Mid-life microbiota crises: middle age is associated with pervasive neuroimmune alterations that are reversed by targeting the gut microbiome. Mol Psychiatry (2020) 25:2567–83. doi: 10.1038/s41380-019-0425-1 31092898

[19] Gareau MG Wine E Rodrigues DM Cho JH Whary MT Philpott DJ. Bacterial infection causes stress-induced memory dysfunction in mice. Gut (2011) 60:307–17. doi: 10.1136/gut.2009.202515 20966022

[20] Denninger JK Smith BM Kirby ED. Novel object recognition and object location behavioral testing in mice on a budget. J Vis Exp (2018) (141):10.3791/58593. doi: 10.3791/58593 PMC6800058 30531711

[21] Brombacher TM Nono JK De Gouveia KS Makena N Darby M Womersley J. IL-13-Mediated regulation of learning and memory. J Immunol (2017) 198:2681–8. doi: 10.4049/jimmunol.1601546 28202615

[22] Derecki NC Cardani AN Yang CH Quinnies KM Crihfield A Lynch KR. Regulation of learning and memory by meningeal immunity: a key role for IL-4. J Exp Med (2010) 207:1067–80. doi: 10.1084/jem.20091419 PMC2867291 20439540

[23] Nono JK Ndlovu H Abdel Aziz N Mpotje T Hlaka L Brombacher F. Host regulation of liver fibroproliferative pathology during experimental schistosomiasis *via* interleukin-4 receptor alpha. PloS Negl Trop Dis (2017) 11:e0005861. doi: 10.1371/journal.pntd.0005861 28827803 PMC5578697

[24] Abdel Aziz N Nono JK Mpotje T Brombacher F. The Foxp3+ regulatory t-cell population requires IL-4Ralpha signaling to control inflammation during helminth infections. PloS Biol (2018) 16:e2005850. doi: 10.1371/journal.pbio.2005850 30379806 PMC6231676

[25] Collins SM Surette M Bercik P. The interplay between the intestinal microbiota and the brain. Nat Rev Microbiol (2012) 10:735–42. doi: 10.1038/nrmicro2876 23000955

[26] Erny D Hrabe de Angelis AL Jaitin D Wieghofer P Staszewski O David E. Host microbiota constantly control maturation and function of microglia in the CNS. Nat Neurosci (2015) 18:965–77. doi: 10.1038/nn.4030 PMC5528863 26030851

[27] Wang T Hu X Liang S Li W Wu X Wang L. Lactobacillus fermentum NS9 restores the antibiotic induced physiological and psychological abnormalities in rats. Benef Microbes (2015) 6:707–17. doi: 10.3920/BM2014.0177 25869281

[28] Sarkar A Harty S Lehto SM Moeller AH Dinan TG Dunbar RIM. The microbiome in psychology and cognitive neuroscience. Trends Cognit Sci (2018) 22:611–36. doi: 10.1016/j.tics.2018.04.006 29907531

[29] Kang M Ryu H-H Lee Y-S. Comparisons of behavior and synaptic plasticity among three C57BL/6 substrains. Anim Cells Syst (2015) 19:181–7. doi: 10.1080/19768354.2015.1023830

[30] Schretter CE Vielmetter J Bartos I Marka Z Marka S Argade S. A gut microbial factor modulates locomotor behaviour in drosophila. Nature (2018) 563:402–6. doi: 10.1038/s41586-018-0634-9 PMC6237646 30356215

