# Supplementary material for: Environmental and microbial factors influence affective and cognitive behavior in C57BL/6 sub-strains
Source: Front Immunol. 2023 Apr 25;14:1139913. doi: 10.3389/fimmu.2023.1139913 (PMC10166845; doi:10.3389/fimmu.2023.1139913)
Supplement: Supplementary file 1 [file DataSheet_1.pdf]

Environmental and microbial factors influence affective and cognitive behavior in C57BL/6 sub-strains

Nada Abdel Aziz[1,2,3*], Inssaf Berkiks[1,3], Paballo Mosala[1,3], Tiroyaone M Brombacher[1,3], Frank Brombacher [1,3*]

[1]Cytokine and Disease Group, International Centre for Genetic Engineering and Biotechnology, Cape Town Component, Division of Immunology, Institute of Infectious Diseases and Molecular Medicine, Faculty of Health Sciences, University of Cape Town, Cape Town, South Africa. [2]Immuno-Biotechnology Group, Biotechnology Department, Faculty of Science, Cairo University, Cairo, Egypt. [3]Wellcome Centre for Infectious Diseases Research in Africa, Institute of Infectious Diseases and Molecular Medicine (IDM), Faculty of Health Sciences, University of Cape Town, Cape Town 7925, South Africa.

# These authors contributed equally

***Correspondence: frank.brombacher@icgeb.org (F. Brombacher) and nadaabdelaziz@cu.edu.eg (N. Abdel Aziz)**

Supplementary Figures Legend

Fig S1. Gating strategies. (A) Gating strategy for identifying cytokine producing CD4$^+$ T cells in meninges. **(B)** Gating strategy for identifying cytokine producing CD11b$^+$ myeloid cells in meninges. **(C)** Gating strategy for identifying cytokine-producing microglia in hippocampus.

Fig S2. Cognitive behaviour in C57BL/6 sub-strains. (A) Exploration ratio and **(B)** Discrimination index in NORT were calculated in C57BL/6N and C57BL/6J upon FMT. **(C)** Exploration ratio, **(D)** Discrimination index, and **(E)** Total exploration time being depicted after long-term co-housing. **(F)** Exploration ratio and **(G)** Discrimination index in OLT were measured in C57BL/6 sub-strains upon FMT. **(H)** Exploration ratio and **(I)** Discrimination index in OLT were measured after long-term co-housing. Results are representative of two

independent experiments with 5–7 mice/group. Data are expressed as mean ± S.E.M. NS, $P > 0.05$; * $P < 0.05$, ** $P < 0.001$, *** $P < 0.0001$ by two-tailed unpaired Student t test.

Fig S3. meningeal CD11b$^+$ myeloid cells in C57BL/6 sub-strains. (A) Frequency of CD11b$^+$ cells and **(B)** IL-4, **(C)** IL-5, **(D)** IL-6, and **(E)** IL-13-expressing CD11b$^+$ cell in C57BL/6N and C57BL/6J upon FMT. **(F)** Frequency of CD11b$^+$ cells and **(G)** IL-4, IL-6, and IL-10-expressing CD11b$^+$ cell in C57BL/6 sub-strains after long-term environmental co-housing. Results are representative of three independent experiments with 5–7 mice/group. Data are expressed as mean ± S.E.M. NS, $P > 0.05$; * $P < 0.05$, ** $P < 0.001$, *** $P < 0.0001$ by two-tailed unpaired Student t test.

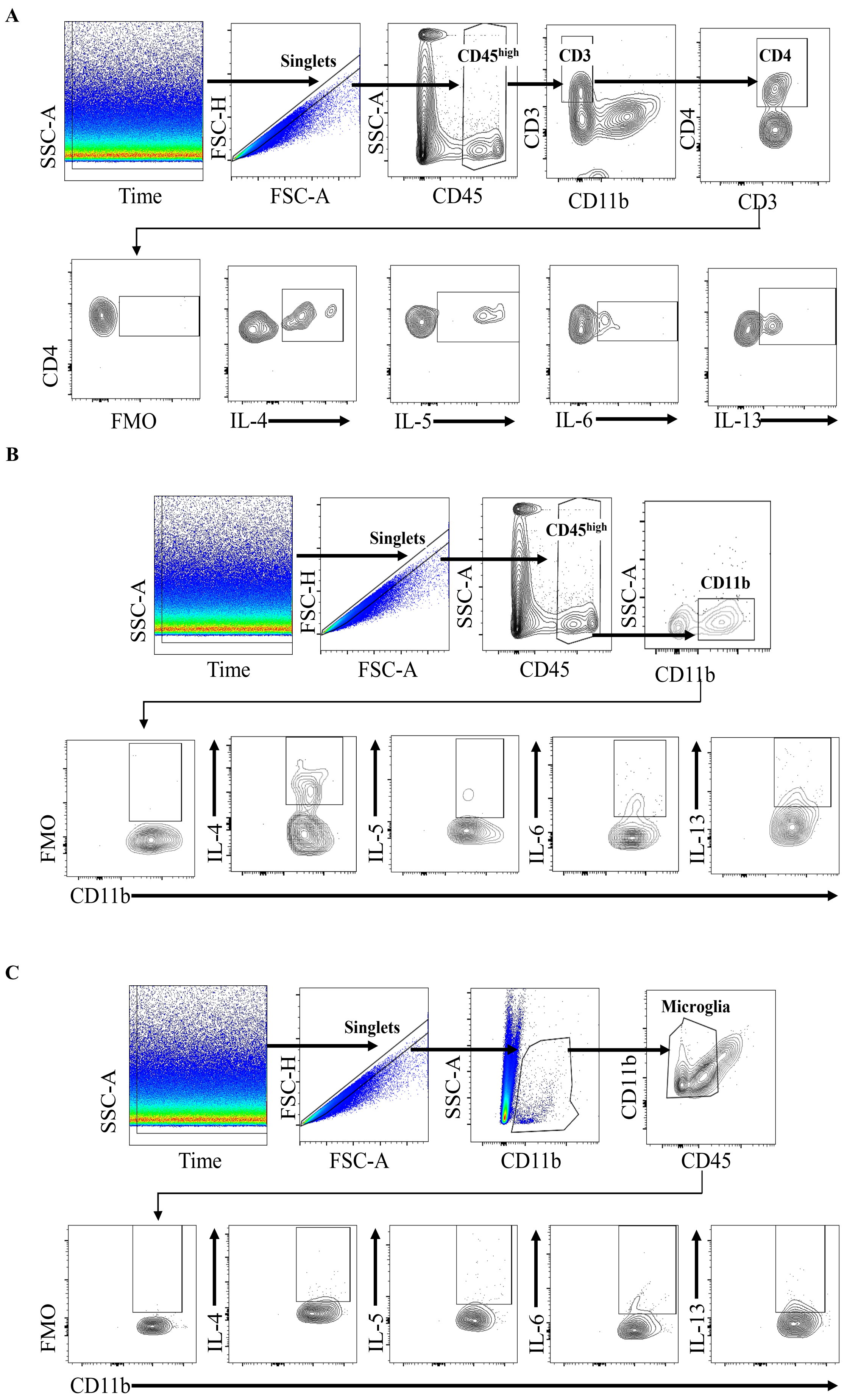

Fig S1. Gating strategies. (A) Gating strategy for identifying cytokine-producing $CD4^+$ T cells in meninges. **(B)** Gating strategy for identifying cytokine-producing $CD11b^+$ myeloid cells. **(C)** Gating strategy for identifying cytokine-producing microglia.

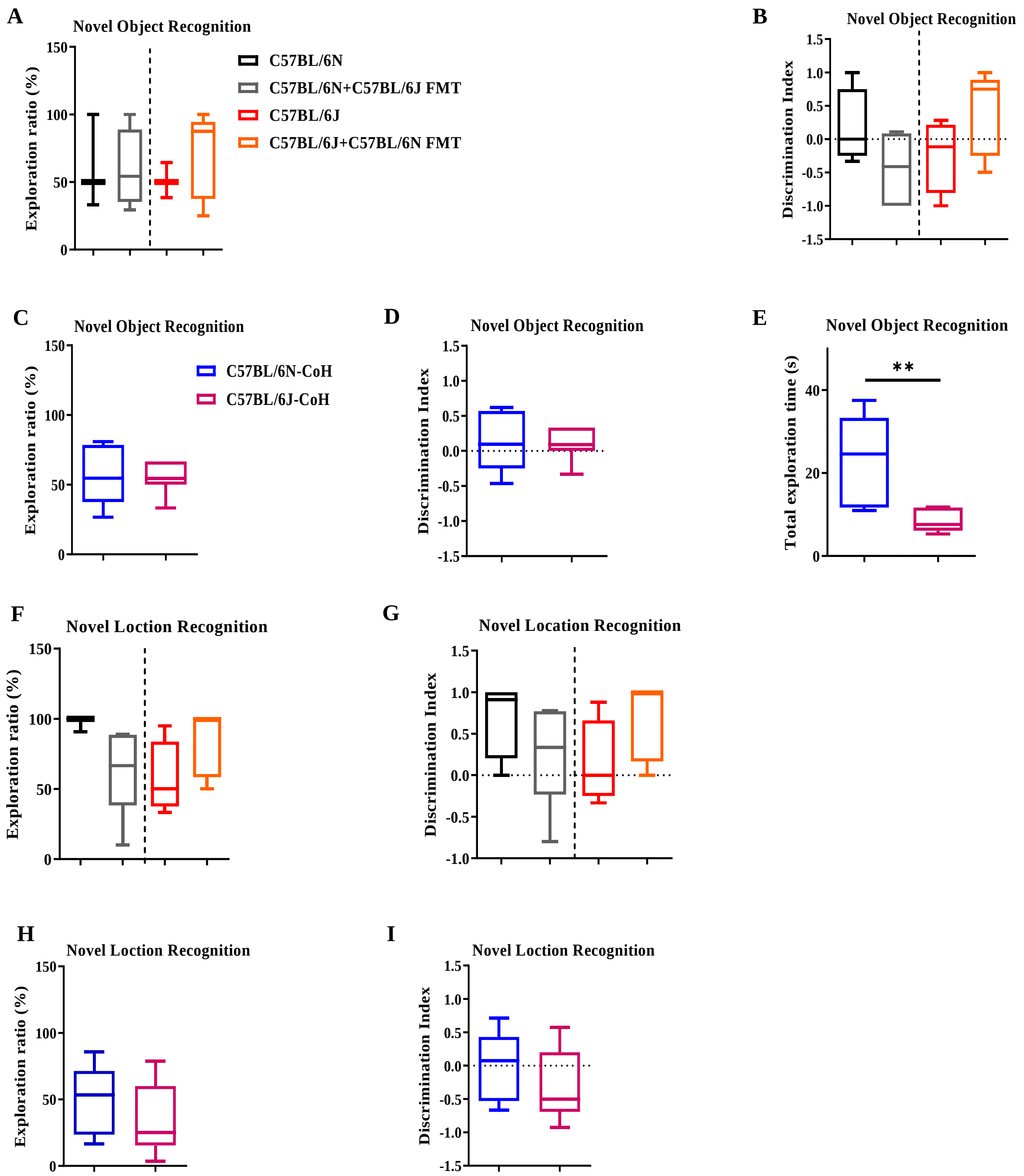

Fig S2. Cognitive behaviour in C57BL/6 sub-strains. (A) Exploration ratio and **(B**) Discrimination index in NOR were calculated in C57BL/6N and C57BL/6J upon FMT. **(C)** Exploration ratio, **(D)** Discrimination index, and **(E) T**otal exploration time being depicted after long-term co-housing. **(F)** Exploration ratio and **(G)** Discrimination index in OLT were measured in C57BL/6 sub-strains upon FMT. **(H)** Exploration ratio and **(I)** Discrimination index in OLT were measured after long-term co-housing. Results are representative of two independent experiments with 4–7 mice/group. Data are expressed as mean ± S.E.M. ** P < 0.001 by two-tailed unpaired Student t test.

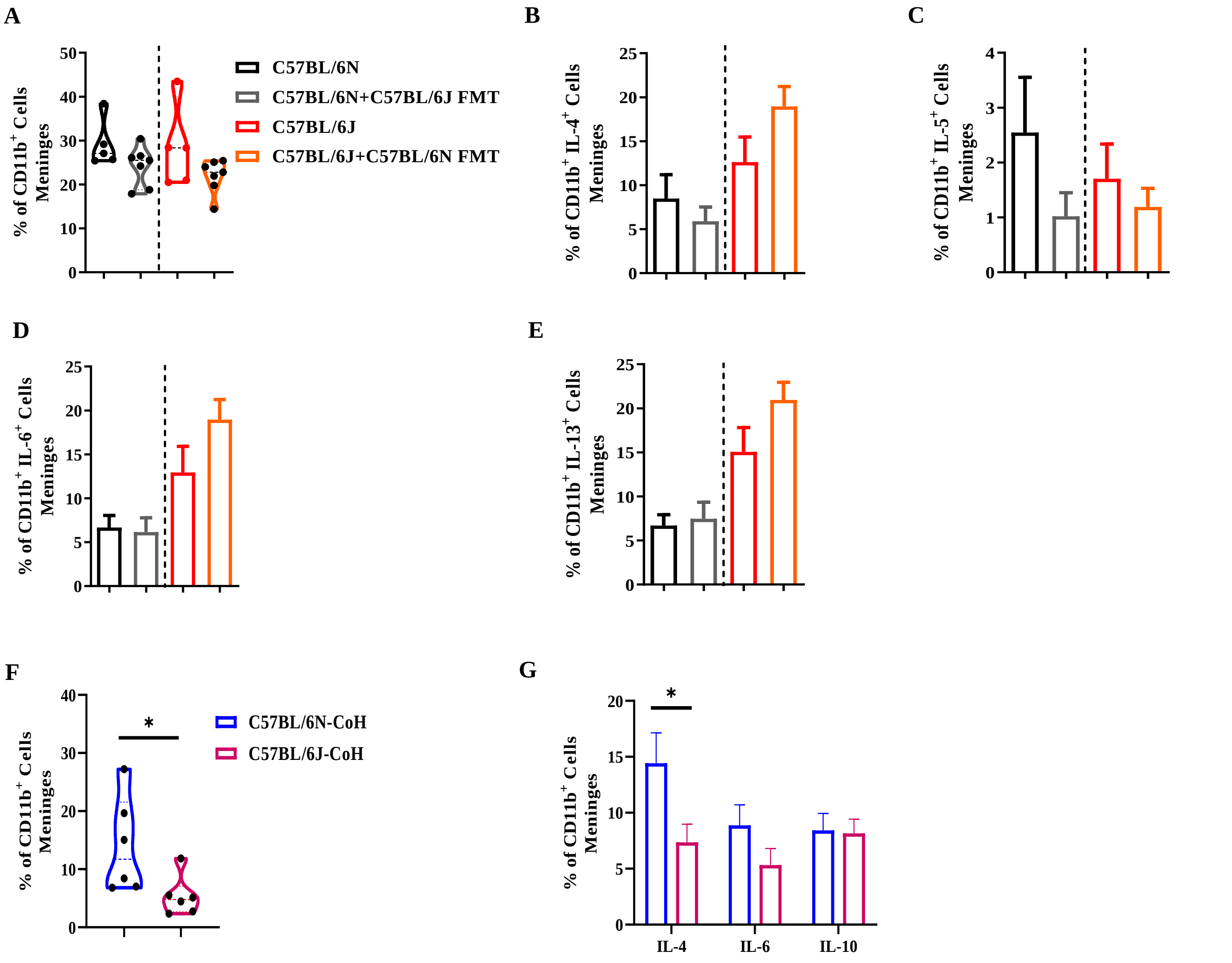

Fig S3. meningeal CD11b⁺ myeloid cells in C57BL/6 sub-strains. (A) Frequency of CD11b⁺ cells and **(B)** IL-4, **(C)** IL-5, **(D)** IL-6, and **(E)** IL-13-expressing CD11b⁺ cell in C57BL/6N and C57BL/6J upon FMT. **(F)** Frequency of CD11b⁺ cells and **(G)** IL-4, IL-6, and IL-10-expressing CD11b⁺ cell in C57BL/6 sub-strains after long-term environmental co-housing. Results are representative of three independent experiments with 5–7 mice/group. Data are expressed as mean ± S.E.M. * $P < 0.05$ by two-tailed unpaired Student t test.
